# A Comprehensive ddPCR Strategy for Sensitive and Reliable Monitoring of CAR-T Cell Kinetics in Clinical Applications

**DOI:** 10.3390/ijms25168556

**Published:** 2024-08-06

**Authors:** Gertrud Wiedemann, Ulrike Bacher, Raphael Joncourt, Françoise Solly, Corinne C. Widmer, Sacha Zeerleder, Urban Novak, Thomas Pabst, Naomi A. Porret

**Affiliations:** 1 Department of Hematology and Central Hematological Laboratory, Inselspital, Bern University Hospital, University of Bern, 3010 Bern, Switzerland; gertrud.wiedemann@insel.ch (G.W.); veraulrike.bacher@insel.ch (U.B.); raphael.joncourt@insel.ch (R.J.); 2 Department for BioMedical Research, University of Bern, 3008 Bern, Switzerland; sacha.zeerleder@luks.ch; 3 Service and Central Laboratory of Hematology, Lausanne University Hospital (CHUV), 1011 Lausanne, Switzerland; francoise.solly@chuv.ch; 4 Department of Medical Oncology and Hematology, University Hospital Zurich, 8091 Zurich, Switzerland; corinne.widmer@usb.ch; 5 Department of Hematology, University Hospital of Basel, 4031 Basel, Switzerland; 6 Laboratory Medicine, Diagnostic Hematology, 4031 Basel, Switzerland; 7 Department of Medical Oncology, Inselspital, University Hospital of Bern, 3010 Bern, Switzerland; urban.novak@insel.ch (U.N.); thomas.pabst@insel.ch (T.P.); 8 Center for Hemato-Oncology, University Cancer Center, 3010 Bern, Switzerland

**Keywords:** droplet digital PCR (ddPCR), CAR-T cell therapies, molecular monitoring, B-cell lymphoma, acute lymphatic leukemia (ALL)

## Abstract

In this study, we present the design, implementation, and successful use of digital droplet PCR (ddPCR) for the monitoring of chimeric antigen receptor T-cell (CAR-T) expansion in patients with B-cell malignancies treated with different CAR-T products at our clinical center. Initially, we designed a specific and highly sensitive ddPCR assay targeting the junction between the 4-1BB and CD3ζ domains of tisa-cel, normalized with *RPP30*, and validated it using blood samples from the first tisa-cel-treated patient in Switzerland. We further compared this assay with a published qPCR (quantitative real-time PCR) design. Both assays showed reliable quantification of CAR-T copies down to 20 copies/µg DNA. The reproducibility and precision were confirmed through extensive testing and inter-laboratory comparisons. With the introduction of other CAR-T products, we also developed a corresponding ddPCR assay targeting axi-cel and brexu-cel, demonstrating high specificity and sensitivity with a limit of detection of 20 copies/µg DNA. These assays are suitable for CAR-T copy number quantification across multiple sample types, including peripheral blood, bone marrow, and lymph node biopsy material, showing robust performance and indicating the presence of CAR-T cells not only in the blood but also in target tissues. Longitudinal monitoring of CAR-T cell kinetics in 141 patients treated with tisa-cel, axi-cel, or brexu-cel revealed significant expansion and long-term persistence. Peak expansion correlated with clinical outcomes and adverse effects, as is now well known. Additionally, we quantified the CAR-T mRNA expression, showing a high correlation with DNA copy numbers and confirming active transgene expression. Our results highlight the quality of ddPCR for CAR-T monitoring, providing a sensitive, precise, and reproducible method suitable for clinical applications. This approach can be adapted for future CAR-T products and will support the monitoring and the management of CAR-T cell therapies.

## 1. Introduction

In 2017, the FDA approved a chimeric antigen receptor (CAR) T cell therapy for the first time, tisa-cel (tisagenlecleucel, Kymriah) targeting CD19. In the same year, axi-cel (axicabtagene ciloleucel, Yescarta) was approved as the second CAR-T immunotherapy product. These two products soon became an important option for the therapy of B-cell malignancies, relapsed or refractory (r/r) large B-cell lymphoma including diffuse large B-cell lymphoma (DLBCL) after two or more prior lines of therapy and B-ALL (B-lineage acute lymphoblastic leukemia) in pediatric or young patients. In February 2021, liso-cel (lisocabtagene maraleucel, JCAR017, Breyanzi) was FDA approved for the treatment of r/r large B-cell lymphoma in adult patients, followed in October 2021 by brexu-cel (brexucabtagene autoleucel, KTE-X19, Tecartus), a product with identical sequence to axi-cel, but a different production process including T-cell enrichment, for the treatment of r/r mantle cell lymphoma in adult patients. The selection of CD19 as the target was made even if it was not a tumor-specific antigen as it is highly expressed on most malignant B-cells, but not on hematopoietic stem cells, limiting the risk of aplastic anemia [1].

Early in the development of CAR-T therapies, researchers identified the B-cell maturation antigen (BCMA) as a promising target for the treatment of relapsed/refractory (R/R) multiple myeloma in addition to the CD19-directed CAR-Ts, which target B-cell malignancies. At present, two anti-BCMA-directed CAR-T products are FDA approved for the treatment of extensively pretreated patients with r/r multiple myeloma after four or more prior therapies, namely ide-cel (idecabtagene vicleucel, bb2121, Abecma) and cilta-cel (ciltacabtagene autoleucel, LCAR-B38M, JNJ-4528, JNJ-68284528) [2,3]. In addition to the already FDA-approved CAR-T products, a large number of trials has pursued the development of new-generation CAR-Ts with improved efficacy and reduced toxicity, targeting a wide variety of antigens that are potential therapeutic options for myeloma, myeloid malignancies, T cell malignancies, and even solid tumors [4].

In the pivotal studies for approval by the medical agencies, substantial survival benefits, or even possible cure for patients is described for the different commercially available products [2,3,5,6,7,8]. These findings are confirmed by numerous subsequent studies as well as in real-life clinical settings post approval [9,10,11,12,13,14,15]. In short, long-term durable remission was demonstrated in various studies with different patient groups receiving CAR-T cells, with durable remission and prolonged survival in ~40–60% of patients treated for B-cell lymphoma as well as ~80–90% in pediatric or young patients with B-ALL as summarized by others [15,16]. 

After Swiss approval of the first commercial CAR-T product in October 2018, the first patient in Switzerland was treated with tisa-cel for relapsed B-ALL at the Inselspital in Bern. A recently published study put the number of Swiss patients aged over 30 who received CAR-Ts for treatment of DLBCL from November 2018 to June 2021 at 81 [17], with 50 of them at our center. At the time of this report (06/2024), around 200 patients received CAR-T treatment at our center, 141 of them with the commercially available products tisa-cel, axi-cel, or brexu-cel. 

Numerous studies have shown that CAR-T cell expansion and persistence in vivo strongly correlate with treatment success. Therefore, these parameters are considered relevant biomarkers for the monitoring of patients following CAR-T treatment, in addition to traditional imaging strategies. Generally, as described in the literature, and also observed in the patients treated in our center, CAR-T cells in the patients’ peripheral blood reach peak levels between 1 and 2 weeks after infusion, with a significant correlation of transgene copy number to response, and can persist up to several years [18,19,20,21]. For the detection of CAR-T cells and monitoring of their kinetics in vivo, different approaches and bioanalytical platforms can be utilized, including the following: for direct quantification, flow cytometry and quantitative PCR; and for assessing the indirect effects of CAR-Ts, a variety of immunoassays for the detection of cytokines like interleukins, interferons, and tumor necrosis factors. Each platform has its advantages and disadvantages as well as its area of application as follows: Flow cytometry is a tool which is suitable for establishment in a routine laboratory setting. As cells are used for the measurement, it allows not only monitoring of early CAR-T cell expansion but also characterization of the CAR-T cell phenotype. The quantitative PCR methods, real-time PCR and ddPCR, can also be easily established in appropriately equipped laboratories, especially ddPCR, a very robust and sensitive tool for longitudinal molecular monitoring of CAR-T copy numbers using DNA as a template [22,23,24,25,26]. After CAR-T infusion, large quantities of cytokines are produced, and some cytokine signatures may enable the prediction of adverse effects; on the other hand, levels are also impacted by confounding factors like sepsis, degree of CAR-T cell expansion, and tumor burden. The lack of validated fast quantification methods is a major limitation in clinical practice [24].

CAR-T cells can cause adverse side effects, namely cytokine release syndrome (CRS) and immune effector cell-associated neurotoxicity syndrome (ICANS), previously known as cytokine release encephalopathy syndrome (CRES), due to inflammatory cytokines and the subsequent receptor–antigen interaction with their target cells. Several studies have suggested a correlation of certain patterns of CAR-T expansion in the peripheral blood to the development of CRS and ICANS [5,27,28,29,30,31].

It is accepted that monitoring CAR-T expansion and persistence in the peripheral blood facilitates the individual prediction of treatment success and assessment of risk stratification for the development of adverse side effects. This approach allows for timely therapeutic intervention. Consequently, there is a growing focus on developing suitable laboratory assays for monitoring CAR-T cell concentration in the peripheral blood alongside clinical monitoring. Molecular methods for the quantification of CAR-T copy numbers on DNA level were initially established using real-time PCR. There are different approaches for CAR-T specific molecular assay design, such as the following: either by targeting the junction region between the CD8 transmembrane region and the CD3ζ signaling chain [32], or the FMC63-28Z gene of the single-chain variable fragment (scFv) not being specific but the sequence is identical for tisa-cel and axi-cel [33,34,35] or the vector backbone [36,37].

Several reference genes have been described as targets for this purpose, including *RPP30* (Ribonuclease P/MRP Subunit P30), *GAPDH* (Glyceraldehyde 3-phosphate dehydrogenase), or the non-transcribed genomic sequence upstream of the CDKN1A/p21 (cyclin-dependent kinase inhibitor 1A), *RPPH1* (Ribonuclease P RNA Component H1), and *TERT* (telomerase) [32,33,34]. Various strategies have been published for quantifying and reporting these results, such as copies CAR-T/500 ng template DNA, the ratio of copies CAR-T/µL to copies reference gene/µL [33], and copies CAR-T/µg DNA, with the latter recommended since 2020 in the FDA guidelines [32,34,38,39]. Several recent studies have described the use of droplet digital PCR (ddPCR) for detection and quantification of CAR-Ts, including the following: In the study by Lou et al. the two platforms, real-time PCR and ddPCR, were compared for CAR transgene copy number quantification, targeting the scFv of the CARs and using RNase P protein *POP4* as a reference [40]. They demonstrated, as many other studies have, that the ddPCR method, which allows for absolute quantification without the need for external references and standard curves, performed better than the real-time PCR in terms of repeatability, reproducibility, and lower limit of detection. In the approach described by Fehse et al. [37], quantification for axi-cel is based on the mean vector copy number and fraction of CAR-T positive cells of all PBMCs, hence the result is given as CAR-T positive cells/µL; three different assays targeting the vector sequence were compared. In a further step, they developed a universal ddPCR assay for the monitoring of tisa-cel as well as axi-cel CAR-T cells in vivo, by targeting a sequence of the FMC63 part, which is identical in both vectors, and using the hematopoietic cell kinase (HCK) gene as reference [35].

When the first patient in Switzerland received CAR-T treatment in 2019, our aim was the design and implementation of a sensitive, specific, and reproducible method for CAR-T copy number quantification in the routine setting to facilitate the monitoring of CAR-T expansion from various sample types in patients with B-cell malignancies treated with different commercially available CAR-T products at our clinical center. By additionally applying this ddPCR method for the quantification of CAR-T mRNA, we aimed to study if the CAR-T transgene expression matches the longitudinal patterns of copy numbers measured on DNA level.

## 2. Results

### 2.1. Approach and Strategy

In this manuscript, we describe the ddPCR strategy developed at our center for the in vivo monitoring of CAR-T cell concentration in peripheral blood and other sample types following the infusion. When CAR-T therapy was introduced in Switzerland, there were no commercially available assays for copy number quantification and all published strategies were real-time PCR based. We developed a strategy allowing for absolute quantification by specific ddPCR assays for all commercially available CAR-T cell products targeting CD19 that are currently approved in Switzerland for B-cell malignancies (tisa-cel, liso-cel, axi-cel, and brexu-cel). For each CAR-T cell product, we applied the same strategy as follows: the specific assay targets the sequence of the intracellular junction between the effector 4-1BB (CD137) or CD28, respectively, and the costimulatory CD3ζ domain. *RPP30* is used as reference gene for normalization to allow for the reporting of the quantitative result as copies CAR-T/µg DNA, as specified by the FDA in the relevant guidelines [39]. Unlike the vector-based quantification methods cited above, our strategy allows for the quantification of the actual CAR-T copies available for expression in the patient, independent of the clonal evolution of the initially polyclonal CAR-T product from infusion over time.

Our assay design was based on published nucleotide sequences when available, such as for tisa-cel [41]. If the exact nucleotide sequence was unknown, we would use the amino acid sequence from the relevant patent for database searches and subsequent Sanger sequencing. It should be noted that the nucleotide sequence can vary between different constructs, even when the same domains are used. Therefore, knowing the exact nucleotide sequence is crucial for the designing and establishing of a product-specific assay. The nucleotide sequence of the CD3ζ domain is the same for tisa-cel and axi-cel/brexu-cel, but different from liso-cel, as Sanger sequencing has revealed.

We quantified the CAR-T copy numbers longitudinally in samples of peripheral blood taken from patients after receiving the treatment. For tisa-cel, we performed a head-to-head comparison of the published real-time PCR assay [32], adapted to ddPCR with our own previously published, in-house-designed assay, which also includes a liso-cel targeting assay [31]. If available, a dilution series of positive cells for the respective CAR-T construct was measured to determine the sensitivity of each assay. CAR-T was also quantified by ddPCR from bone marrow aspirates, biopsies, or other material sources taken at selected time points and compared to the results from peripheral blood. Furthermore, the ddPCR method was used to investigate if and to what extent actual CAR-T expression, measured by ddPCR from mRNA, can be inferred from the CAR-T copy numbers. As tisa-cel was the first commercially available CAR-T product in Switzerland, we conducted an inter-laboratory comparison of the ddPCR-based CAR-T copy number quantification with selected patient samples at two other laboratories to ensure reliable performance; for axi-cel and liso-cel, the comparison was performed at one other molecular diagnostic laboratory in Switzerland.

### 2.2. Patient Cohort

Our patient cohort included 141 patients receiving commercially available CAR-T products approved by Swissmedic, with 73 patients treated with tisa-cel for B-cell lymphomas or ALL, 50 with axi-cel for B-cell lymphomas, and 18 with brexu-cel for mantle cell lymphoma or ALL (Table 1). The period ranged from when the first patient started receiving tisa-cel for the treatment of ALL in January 2019 to May 2024.

### 2.3. Assay Development and Initial Testing

#### 2.3.1. Tisa-Cel

As the first patient receiving CAR-T therapy in Switzerland was treated with tisa-cel, this was the first assay established and thoroughly tested in our laboratory. At this time, there were no such assays commercially available, and all the strategies published at that time were real-time PCR based. As previously described [31], we designed a tisa-cel sequence-specific assay and designed a ddPCR-adapted version of the real-time PCR assay published by Milone et al. [32]. In a first step, using the blood samples from the first tisa-cel treated patient, we compared the performance of these two ddPCR assays. They both target the same region, but employ different nucleotide sequences for primers and probes, specifically covering the junction between the CD8 transmembrane region and the ζ signaling chain. The proper cluster separation of positive and negative droplets was assessed in the graphical representation of the results in the 1D and 2D plots (Supplemental Figure S1A–C). The level of blank (LOB) is shown in Supplemental Table S1. For our self-designed ddPCR assay, no droplet was positive for the CAR-T-specific PCR product in 48 wells of WT genomic DNA. For the ddPCR assay, using the sequence published by Milone et al. [32], 1 CAR-T positive droplet was measured in 6 of the 100 analyzed wells. The sensitivity and correlation of the two tisa-cel assays were determined using a dilution series of DNA (described in the methods and shown in Supplemental Figure S1D). For both these assays, 20 copies of CAR-T/µg of DNA could be reliably quantified when enough input template was used for the assay, which corresponds to a *RPP30* value of at least 1136 copies/µL measured for the sample. It is possible to detect lower copy numbers, but due to the small number of CAR-T positive droplets in such samples, detection and quantification is not reliable. Therefore, for both tisa-cel specific assays, we set a limit of detection of 20 copies CAR-T/µg DNA as the sensitivity of the ddPCR method (Supplemental Figure S1D).

**Figure 1:**
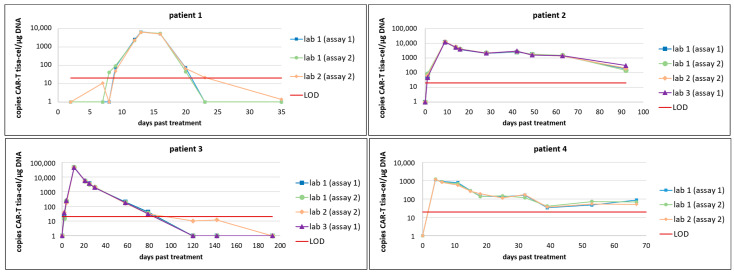
Comparison of CAR-T tisa-cel quantification using ddPCR with two different assays in up to three different laboratories. For 4 different tisa-cel treated patients (patient 1: B-ALL; patients 2, 3 and 4: r/r DLBCL), the CAR-T copy number was quantified in different laboratories using the self-designed assay (assay 1) or the assay according to Milone et al. [32] (assay 2). Lab 1: Inselspital, Bern University Hospital, lab 2: CHUV (Centre Hospitalier Universitaire Vaudois, Lausanne), lab 3: USZ (University Hospital Zürich).

Next, we selected consecutive patient samples from four patients treated with tisa-cel and analyzed them in parallel with the two assays. The qualitative results matched for 50 of 51 samples, and the quantitative values were consistently comparable over time (Figure 1, Supplemental Table S2).

After a thorough evaluation of the parameters’ sensitivity and correlation, the reproducibility and precision of the two tisa-cel specific assays were confirmed in our laboratory by measuring several patient samples in independent experiments. Subsequently, a set of patient samples was analyzed in an inter-laboratory exchange (see the paragraph on inter-laboratory comparison below).

#### 2.3.2. Axi-Cel/Brexu-Cel

For the axi-cel CAR-T cell product, our assay design was based on the published nucleotide sequence of the construct [41] targeting the CD28 and CD3ζ domains, with the probe covering the junction and validated as described above for tisa-cel. As the sequence for brexu-cel is identical to that of axi-cel, the same assay was used for the CAR-T copy number quantification for both products. For the axi-cel/brexu-cel-specific assay of WT genomic DNA, there was 0 copies/µg DNA in 68 wells (no CAR-T positive droplets were detected in any of the wells, Supplemental Table S3). There was a clear cluster separation of positive and negative droplets using the 1D and 2D plots (Supplemental Figure S2A,B). The sensitivity and correlation of this assay, using serial dilutions of DNA extracted from the CAR-T positive cells and two patient samples, are shown in Supplemental Figure S2C. Based on these results, 20 copies of CAR-T/µg DNA can be detected and quantified reliably with the axi-cel/brexu-cel-specific assay, if a minimal amount of *RPP30* copies of 1136 copies/µL is reached in the ddPCR. The parameters’ reproducibility and precision were analyzed as described above. Five selected positive patient samples were analyzed in two additional Swiss laboratories (see the paragraph inter-laboratory comparison below).

#### 2.3.3. Inter-Laboratory Comparison of ddPCR Assay Performance

When the molecular monitoring of CAR-T kinetics was established in several Swiss centers, a comparison of ddPCR assay performance was carried out to study the reproducibility of the method in different laboratories. Therefore, selected longitudinal samples from four patients from our comparison of the two tisa-cel specific assays were analyzed by ddPCR in two additional laboratories in Switzerland. The qualitative and quantitative results are highly comparable between the two tisa-cel specific assays and across the three different locations, laboratory 1, 2, and 3 (Figure 1, Supplemental Table S2).

The CAR-T kinetics in patient 1, receiving tisa-cel for the treatment of B-ALL, showed rapid expansion at day 8 (assay 2, lab 1) to 9 (assay 1, lab 1 and assay 2, lab 2), followed by a rapid disappearance of CAR-Ts in the peripheral blood three weeks post-treatment. The expansion patterns measured in the different laboratories using the two different assays for patients 2, 3 and 4, treated with tisa-cel for r/r DLBCL, showed a rapid CAR-T expansion already above the level of detection at day 1 post treatment (patient 2), and different levels of peak expansion with over 45,000 copies/µg DNA in patient 3 at day 11 compared to around 1150 copies/µg DNA in patient 4 at day 4 After the peak of expansion, a slow decrease and persistence is observed in patients 2 and 4, while in patient 3, after the rapid and high peak expansion, the copy number drops with each longitudinal sample until the last positive sample at day 79 post treatment; all subsequent measurements are negative (below the LOD depicted as red line, Figure 1).

For axi-cel and liso-cel, selected patient samples were also measured in parallel at two sites with highly comparable quantitative results for axi-cel (Supplemental Figure S3 and Table S4) and liso-cel with concordant results (according to Bland-Altman) and a linear relationship (correlation tests, Passing Bablock) (Supplemental Table S5 and Figure S4).

#### 2.3.4. Analysis from Other Materials Than Blood Samples

**Figure 2:**
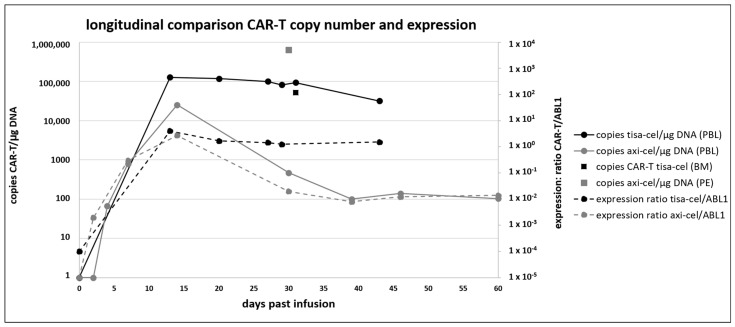
Longitudinal comparison of CAR-T copy numbers and expression. CAR-T copy number (solid line) and expression (dotted line) quantified from one tisa-cel (black) and one axi-cel (grey) treated patient. Additionally, for the tisa-cel treated patient a sample from bone marrow (BM) was analyzed and for the axi-cel treated patient pleural effusion (PE).

The method for CAR-T copy number quantification by ddPCR can be successfully applied to various sources of material apart from peripheral blood. These sources include bone marrow, FFPE, or native biopsy material from lymph nodes, pleural infusion, and bronchoalveolar lavage (Figure 2, Supplemental Table S6). As with other ddPCR applications, this robust method works reliably with DNA extracted from a wide range of materials, even when the amount or quality of the input material is suboptimal. In such cases, the sensitivity of the assays might be reduced if the required number of *RPP30* copies/µL in the ddPCR result is not met. Therefore, the theoretical limit of detection needs to be calculated and reported accordingly.

Comparison of the CAR-T copy number quantification from other materials to the routinely analyzed peripheral blood samples clearly shows the recruitment of circulating CAR-Ts from the peripheral bloodstream to the target tissues like bone marrow and lymph nodes or other sources like pleural effusion (Figure 2, Supplemental Table S6).

Concerning the biopsy samples, native material is preferred over FFPE samples; this can be demonstrated in the tisa-cel-treated patient 12 (Supplemental Table S6), for whom both materials (native material versus FFPE) from the same lymph node biopsy were analyzed. In general, when assaying the FFPE material, the absolute number of CAR-T copies tends to be underestimated due to lower template quality. Still, analysis of the FFPE material from the lymphoma patients clearly shows that CAR-T copies are detectable by this method not only in the peripheral blood, but also in the target tissues, even at higher levels than in the peripheral blood (Supplemental Table S6, patients 12 tisa-cel and 1 axi-cel). This recruitment to target tissues is observed persistently for more than 9 months post CAR-T cell infusion (Supplemental Table S6, patient 12 tisa-cel).

#### 2.3.5. Examples of CAR-T Copy Number Kinetics from Patients

In the patient cohort shown here, the peak of CAR-T expansion measured from blood samples was reached at a mean interval of 12 days post infusion for tisa-cel (n = 73; 2–84 days) and 11 days for axi-cel. For brexu-cel the mean interval to maximal CAR-T expansion in the peripheral blood was at day 17 post CAR-T cell infusion (n = 17; 5–61 days) (Table 1, peak CAR-T expansion day). One patient was excluded because of late expansion at day 171 after induction with glofitamab, as reported previously [42]. In tisa-cel treated patients, the average CAR-T copy number quantified from peripheral blood at the peak level of expansion was 9733 copies CAR-T/µg DNA (n = 72; 265–127,942 copies CAR-T/µg DNA). For axi-cel this average number was 12,375 copies CAR-T/µg DNA (n = 50; 37–91,575 copies CAR-T/µg DNA) and for brexu-cel 7396 (n = 17, 14–92,877 copies CAR-T/µg DNA) (Table 1, peak CAR-T expansion average copy number/µg DNA). These numbers are calculated without taking clinical parameters or any interventions into account (Table 1, peak CAR-T expansion). More than half of all patients from our cohort reached the time points of 6 and 12 months after treatment, as demonstrated by the following numbers: tisa-cel, 66% and 59% (n = 48/43 patients of 73 in total), axi-cel, 84% and 78% (n = 42/39 of 50 in total), and brexu-cel, 78% and 72% (14/13 of 18) (Table 1, survival > 6 months and >1 year).

The longest persistence of CAR-T cell copies observed in patient blood samples in our cohort until now is up to 4.5 years for tisa-cel and axi-cel (55 and 56 months, respectively) and nearly 3.5 years for brexu-cel (42 months). As more time passes since the introduction of CAR-T therapy at our center, we expect to measure even longer persistence in future.

In the patients who reached the time point of 1 year after CAR-T therapy, which the CAR-T copy number was analyzed until, 33 out of 34 (97%) showed measurable persistence of tisa-cel, along with 5 out of 6 patients (83%) of brexu-cel, but only 10 out of 21 (48%) of the axi-cel-treated patients (Table 1, persistence > 1 year).

#### 2.3.6. CAR-T mRNA Expression in Comparison to Copy Number Quantification

To study CAR-T transcript expression in vivo in comparison to DNA-based copy number quantification, mRNA expression was quantified longitudinally for seven selected patients following the tisa-cel as well as axi-cel treatment, using the same CAR-T-specific ddPCR assay as for DNA-based copy number quantification and *ABL1* as control gene, as previously described by us [43] (Figure 2, Supplemental Table S7).

Figure 2 demonstrates the longitudinal comparison between copy number quantification (solid line) and expression (dotted line) for one tisa-cel (black color)- and one axi-cel (grey color)-treated patient. For the tisa-cel-treated patient, the peak expansion of CAR-T copy number was measured at day 13 past infusion, with 127,942 copies/µg DNA, at the same time point as the peak of expression, with a ratio of 4.037 CAR-T/*ABL1*. Over time, both copy number and expression decreased comparably from these peak values to 31,657 copies/µg DNA and a ratio of 1.521 at day 43. In the example of an axi-cel-treated patient, the peak expansion was reached on day 14, with 25,216 copies/µg DNA and an expression value of 2.711. The next sample, monitored on day 30, showed a clear reduction in CAR-T copies to 465 copies/µg DNA, as well as an expression value of 0.0196. In the subsequent sampling time points at days 39, 46, and 60, the CAR-T cells persisted in the peripheral blood, with copy numbers of 100, 139, and 104 copies axi-cel/µg DNA, respectively. Correspondingly, the expression ratios of CAR-T/*ABL1* were 0.008, 0.012, and 0.014, respectively.

Thus, with this ddPCR approach, CAR-T expression was observed in all the samples in which CAR-T DNA was detected. In longitudinal monitoring, the quantification of the expression and copy numbers matched in terms of magnitude and course of kinetics. Using this methodology for all the samples analyzed, for both CAR-T copy number on the DNA level and expression on the mRNA level, the qualitative results as well as the trends over time were found to correspond well with each other (Figure 2, Supplemental Table S7). The peak values of the mRNA and copy numbers showed a significant correlation, with high peak expression in the peripheral blood associated with more adverse events but not CAR-T cell persistence at 3 months, as already reported [43].

## 3. Discussion

We describe a ddPCR strategy for monitoring CAR-T cell expansion in patients after treatment with the different products in our clinical center used for treatment of DLBCL, mantle cell lymphoma, and ALL. Given the advantages of ddPCR over standard real-time PCR, that include absolute quantification without the need for external standard curves, the ability to quantify targets and references in the same well, improved precision specifically at low target concentrations, high tolerance to PCR inhibitors, and less inter-run variability, this technique was found suitable for monitoring the kinetics of CAR-T cell copies in the recipients of CAR-T cell therapies. The superiority of ddPCR over real-time PCR is well established. For CAR-T monitoring, a direct comparison of both methods demonstrated that the ddPCR approach had better reproducibility, along with higher sensitivity [40,44].

Our strategy for ddPCR monitoring of the CAR-T kinetics in vivo was based on the initial description for copy number quantification of CARs, expressing the CD137 signaling domain by real-time PCR [32]. By targeting the intracellular junction region between the effector 4-1BB (CD137) or CD28, respectively, and the costimulatory CD3ζ domains using sequence-specific assays for each construct, with *RPP30* as a normalizer, an exact and sensitive molecular method for the quantification of CAR-T copy numbers from a variety of patient samples is available for routine monitoring. The assays described here meet the criteria for the limit of quantification of ≤ 50 copies/µg DNA recommended in the 2020 FDA guidelines on long-term follow-up after the administration of human gene therapy products [39]. This approach can also be applied to all new CAR-T products, which will be introduced to the market in the future by designing an appropriate assay for the respective nucleotide sequence of the relevant region of the respective construct. In cases where the nucleotide sequence is not available yet, the nucleotide sequence covering the relevant region of the co-stimulation signaling domain (for example, 4-1BB/CD137 or CD28) and the CD3ζ signaling domain needs to be resolved in a first step. For example, Sanger sequencing, if necessary, with a strategy using ambiguous primers would allow for the design of sequence-specific ddPCR assays for other CAR-T constructs. The methodology was tested thoroughly not only in our laboratory but also through an inter-laboratory comparison, yielding highly comparable results across different sites in terms of limit of detection and copy number quantification from various patient samples.

Performance, reliability, and comparability of two assays with a slightly different nucleotide sequences for primers and probes targeting the same CAR-T domain, namely the junction between the CD8 transmembrane region and the ζ signaling chain of tisa-cel (assay 1 and assay 2), is comparable even when the measurements are performed in different laboratories (Figure 1, Supplemental Table S2). This finding underlines the reliability of target-specific, properly validated ddPCR assays for CAR-T copy number quantification and is a prerequisite for use in routine diagnostics.

By now, different ddPCR-based approaches for monitoring CAR-T transgene levels in patient samples have been reported. Various strategies have been described regarding the targeted CAR-T region and the reference genes selected for normalization. For result quantification, various options are available, such as using the mean vector copy number to calculate the actual number of CAR-T cells per µL blood or determining the fractional abundance of CAR-T and housekeeping gene copies measured in the ddPCR [33,35,37]. Several reports describe clonal kinetics of the initially polyclonal CAR-T cell pool, with the highest clonal diversity in the infusion product decreasing over time during expansion and persistence [45,46,47,48]. Therefore, we hypothesize that our strategy for normalizing and reporting the result as CAR-T copy number per µg of DNA is less affected by the clonal evolution of the product in vivo, compared to a strategy based on the mean vector copy number. Admittedly, this strategy is prone to the underestimation of CAR-T expansion at peak expansion with high copy numbers, as the gDNA originating from CAR-Ts is dominating the extracted gDNA [49]. Nevertheless, as discussed by Sugimoto et al. [44], the current regulatory compliant unit is copies/µg DNA and does not rely on a combination of an assumed transduction rate with a white blood cell count, which might also be inaccurate especially in the lymphodepletion conditions, or a spike-in calibration curve. Additionally, as we are using this method for CAR-T quantification for a variety of different sample types like bone marrow, biopsies (native and FFPE material), and others, a direct comparison to the value from peripheral blood would not be possible when giving the result in a unit like copies/µL of blood. It is known that CAR-T cells are not only circulating in the bloodstream but are also localized at the site of the malignant cells targeted by the CAR-T therapy, like lymph nodes or bone marrow [50]. The method we developed is suitable for the detection of CAR-Ts within a wide variety of sample types, where CAR-Ts are detected in a comparable order of magnitude or even higher than in samples from the peripheral blood.

Initially, in the first case reports, the insertion site of the CAR transgene was suggested as the possible cause for clonal expansion [45,48]. Studies of larger patient cohorts not only identified the potential of insertional mutagenesis for the promotion of therapeutic T-cell proliferation but also a number of additional factors including cell intrinsic properties that might contribute to the differences observed. Transcriptionally distinct clusters with specific expression signatures, namely higher expression of cytotoxicity and proliferation genes, seem to play a key role in this process [46,47]. The extent to which this phenomenon is relevant for changes in CAR-T efficacy over time, irrespective of the number of CAR-T copies available on the DNA level, still needs to be addressed.

We supplemented copy number monitoring of selected CAR-T-treated patients with expression analysis, also quantified by ddPCR using cDNA as template. Assays targeting the vector backbone would not be suitable for this approach, as transcription of the targeted region is essential. In our data, both kinetics showed, longitudinally, the same course with a peak of expansion at the same time, not only on the gDNA but also the mRNA level (Figure 2, Supplemental Table S7). Additionally, the order of decrease in the CAR-T cells in the bloodstream after the initial expansion, concerning time and magnitude as well as persistence over time, correlates at DNA and mRNA level. Thus, by using the sensitive ddPCR method, we show that the transgene sequence is indeed detected not only at the DNA level but is also expressed. According to our results from the longitudinal measurement of both parameters for selected patients, no downregulation of expression compared to the copy number was observed. In the future, the integration of the monitoring of CAR-T expression using ddPCR with cDNA as a template into the routine analysis of CAR-T kinetics is a promising approach to add another dimension to the in vivo monitoring of CAR-Ts, applying the most sensitive molecular methods available.

In the large clinical studies conducted prior to the approval of axi-cel and tisa-cel, data on CAR-T cell expansion and persistence over time on the treatment success differed slightly between the two constructs.

In the pivotal studies for tisa-cel, axi-cel, and brexu-cel, a clear association between the high degree of expansion as well as the duration of persistence of the CAR-T cells detected in the blood stream, quantified with PCR-based methods or peak percentage in flow cytometry, and the response to treatment was shown [8,14,51]. In 2015, Porter had already suggested a grouping according to the peak expansion measured by qPCR or FCM into the following categories: responders with a high maximum expansion, patients achieving partial response with median expansion, and non-responders with minimal expansion [18]. The experience gained from the clinical use of CAR-T cells has confirmed that patients with a high maximum CAR-T expansion are more likely to achieve a favorable response, while in vivo persistence seems to be a less suitable predictor [18,20,25,33,44,52,53]. Additionally, a high peak expansion of CTL019 cells coincides with negative side effects, like the development of immune effector cell-associated neurotoxicity syndrome (ICANS) or cytokine release syndrome (CRS) [26,27,31].

Regarding the differences and similarities of CAR-T kinetics in vivo and outcome, the results of our patient cohort match those of the large pivotal studies and numerous real-life reports currently available [15,26,53,54,55,56]. The copy numbers at peak expansion are higher in the axi-cel-treated patients compared to tisa-cel (12,375 versus 9733, Table 1) in our cohort, while the expansion kinetics are similar (Table 1, Figure 2). Regarding persistence, the results in our small and heterogeneous patient cohort differ in the survivors one year post treatment (Table 1) from the largescale data in the literature reporting no significant difference between the two constructs. We will continue to monitor this parameter as the number of patients treated with CAR-T products continues to increase at our center. With the steadily increasing number of CAR-T-treated patients at our center, in terms of outcome, the slightly better performance of tisa-cel compared to axi-cel is reflected by a higher number of surviving patients at 6 months and 1 year after treatment (84% compared to 66% and 78% to 59%, respectively, Table 1).

In summary, numerous studies and the experience gained from clinical application demonstrate the importance of suitable methods to follow to perform in vivo expansion for the prediction of clinical outcome and early adverse effects. Recent studies favor ddPCR as the more suitable method, at least in the early phase during the first two weeks, compared to flow cytometry with a lower sensitivity and more limited range of detection [25,26]. The ddPCR assays described here, targeting the junction region between the transmembrane and signaling domains, facilitate highly sensitive and robust quantification in vivo, not only of the CAR-T copy numbers but also of their expression over a wide range, from a variety of sample materials.

## 4. Material and Methods

### 4.1. Sample Collection and Preparation

All patients who participated in this study signed the general consent form of the hospital. Ethical procedures were conducted in accordance with Swiss law. The assays described in this manuscript were established for the routine workup of the patients. For the analysis, 5 to 20 mL of peripheral blood was collected in EDTA tubes. After leukocyte enrichment by erythrocyte lysis and centrifugation, one million cells were used for the extraction of genomic DNA using the QIAamp DNA mini kit (Qiagen, Hombrechtikon, Switzerland).

### 4.2. ddPCR

CAR-T cell-specific copy numbers were analyzed with the QX200 droplet digital PCR (ddPCR) system (Bio-Rad, Cressier, Switzerland). Briefly described, the ddPCR reaction was set up using ddPCR Supermix for Probes (no dUTP) (Bio-Rad, Cressier, Switzerland) according to the manufacturer’s instructions with final concentrations of 700 nM for primers and of 200 nM for the probes. For the CAR-T-specific PCR-product, the probe was labelled with FAM, and for the *RPP30* normalizer, HEX. A final amount of 170.8 ng of digested DNA using *Hae*III (New England Biolabs, Ipswich, MA, USA) for all the assays was used as the template in each reaction (20 µL of total volume per well used for droplet generation), and each sample was analyzed in duplicate.

After droplet generation on the AutoDG automated droplet generator (Bio-Rad, Cressier, Switzerland), PCR was performed with 40 cycles for 30 s at 94 °C for denaturation, followed by 1 min at 55 °C for annealing and extension. For readout, the QX200 Droplet Reader (Bio-Rad, Cressier, Switzerland) with the two-color detection system set to FAM (channel 1) and HEX (channel 2) was used.

For the calculation of CAR-T copy numbers per µg of DNA, the instrument’s QuantaSoft software (version 1.7.4, Bio-Rad, Cressier, Switzerland) output of copies per µL for each channel (CAR-T and *RPP30*), merged from the two wells that were analyzed for each sample, was used. One copy of *RPP30* represents one copy of a haploid genome equating to 3.3 pg DNA [57], and the concentration of the DNA in the analyzed sample was calculated as follows: DNA concentration (µg/µL) = *RPP30* concentration from the droplet reader (copies/µL) × 3.3/1 × 10^6^ (adapted from Härmälä et al. [58]). Subsequently, the CAR-T concentration (copies/µL) can be divided using the calculated DNA concentration to obtain the copies of CAR-T/µg of analyzed DNA as follows: CAR-T concentration (copies/µg) = CAR-T concentration from the droplet reader (copies/µL)/*RPP30* DNA concentration (µg/µL).

For expression analysis, RNA was extracted after the initial sample preparation as described above with the QIAamp RNA Blood Mini Kit (Qiagen, Hombrechtikon, Switzerland), followed by the reverse transcription of 2 µg of RNA using the SuperSkriptII RNase H Reverse Transcriptase system (Thermo Fisher Scientific, Basel, Switzerland) with random hexamer primers according to the manufacturer’s protocol. The ddPCR was setup as described above, using cDNA without restriction digest and equivalent to 12.5 ng of the initial input amount of RNA for each well. The result for the expression analysis are given as the ratio, CAR-T/*ABL1*, calculated from the instrument’s QuantaSoft software output of copies per µL for each channel (CAR-T and *ABL1*), merged from the two wells that were measured for each sample.

### 4.3. Design of CAR-T Product Specific Assay Design and Validation

As previously described, we designed sequence-specific primers and probes for the CAR-T constructs, targeting the intracellular junction sequence between the effector 4-1BB (CD137) or CD28, respectively, and the costimulatory CD3ζ domains, as was initially conducted in a real-time PCR approach for the quantification of CARs expressing the CD137 signaling domain [32]. Each assay targets the respective sequences of the intracellular signaling domains of the CAR-T constructs by covering the junction of the co-stimulation signaling domain (4-1BB/CD137 or CD28) and the CD3ζ signaling domain (Table 2 ddPCR assays: primers and probes). A summary of all assays described here, including which CAR-T construct is targeted, as well as the brand name, approval for the treatment of which diseases by Swissmedic, and sensitivity of each assay, is shown in Supplemental Table S8.

Sanger sequencing was performed for PCR products with the same primers. From the first positive patient sample for each CAR-T product, we confirmed that the amplicon indeed targets the intended sequence by conducting Sanger sequencing using primers covering an extended region compared to those used for the ddPCR assay (Table 2 oligonucleotides for PCR and Sanger sequencing).

*RPP30* (ribonuclease P protein subunit 30) was used as normalizer to facilitate the quantification of CAR-T copy numbers per µg DNA as previously described [58]. For expression analysis, a standard qPCR assay for the *ABL1* control gene [59] adapted to our ddPCR settings was used, the result of this mRNA expression analysis is given as a ratio of copies CAR-T/*ABL1*. For all the assays, the CAR-T-specific PCR product is labelled with a FAM fluorophore, the normalizer, *RPP30*, for copy number expression, and *ABL1* for expression analysis, along with HEX. Sequences for all the oligonucleotides used for Sanger sequencing as well as the primer and probe sequences for the ddPCR assays are listed in Table 2.

For tisa-cel, the assay design was based on the sequence given in the patent specification, with a FAM-labelled probe for the detection of the CAR-T-specific PCR product [31]. Additionally, we used the real-time PCR assay published by Milone et al. [32], with exchange of the fluorophore (VIC to FAM) as the only adjustment for ddPCR, to test the comparability of the two different assays. As for each ddPCR assay, the proper cluster separation of positive and negative droplets is assessed in each run using the proper negative and positive controls in the graphical representation of the results in the 1D and 2D plots given by the QuantaSoft software (examples shown in Supplemental Figure S1A–C). If necessary, the threshold for the proper discrimination of positive and negative droplets in channel 1 (FAM) and channel 2 (HEX) needs to be adjusted manually for the whole run using the appropriate negative and positive controls. If no clear visual discrimination between the clusters of negative and positive droplets in one channel is possible, the cycling conditions need to be optimized by running a temperature gradient. The level of blank (LOB) was determined by measuring WT genomic DNA in 48 wells of WT genomic DNA in different runs (Supplemental Table S1). To determine the sensitivity and correlation of the two tisa-cel assays (the self-designed assay and the one designed after Milone et al. [32]), a dilution series of DNA extracted from tisa-cel positive cells covering steps from undiluted to a 1:100,000 dilution in WT genomic DNA was analyzed in two independent runs, each with duplicate wells (Supplemental Figure S1D).

To analyze reproducibility and precision, we selected consecutive samples over time from four patients treated with tisa-cel and analyzed 51 samples in total with the two different assays in parallel (Figure 1, Supplemental Table S2).

For axi-cel and brexu-cel, the assay design was based on the published sequences [41] and validated as described above for tisa-cel. We confirmed that the CAR-T specific amplicon was indeed the target used for the ddPCR assay by Sanger sequencing of the first CAR-T positive blood sample from the first patient receiving axi-cel treatment at our center, using primers covering an extended region. As brexu-cel has a sequence identical to that of axi-cel, the same assay was used for CAR-T copy number quantification for both products. The LOB was measured in 68 wells of WT genomic DNA. The proper cluster separation of positive and negative droplets was assessed as described above 1D and 2D plots (examples shown in Supplemental Figure S2A,B), adjusting the thresholds when necessary and using the proper controls. The sensitivity and correlation of this assay were determined as described above, using serial dilutions of DNA extracted from the CAR-T positive cells and two patient samples with a high number of CAR-T copies. This process covered steps from undiluted to a 1:100,000 dilution in wild-type (WT) genomic DNA, conducted in two independent runs with duplicate wells each (Supplemental Figure S2C). Additionally, the parameters’ reproducibility and precision were analyzed as described above for the tisa-cel assays, as exemplary shown for 6 patient samples (Supplemental Figure S3).

### 4.4. Inter-Laboratory Comparison of ddPCR Assay Performance

For the inter-laboratory comparison, the ddPCR was set up using the standard procedures in each laboratory, resulting in a slightly different DNA input amount per well. Selected samples from our comparison of the two tisa-cel specific assays were analyzed by ddPCR in two additional laboratories in Switzerland, namely the CHUV (Centre Hospitalier Universitaire Vaudois, Lausanne) and USZ (University Hospital Zürich). At CHUV, the assay according to Milone et al. [32] (assay 2) was used, whereas the USZ lab used our self-designed ddPCR assay (assay 1). Twenty-two samples from two patients, with 10 and 12 different time points, respectively, were tested at all locations. An additional 21 samples from two more patients, collected over 11 and 10 different sampling dates, respectively, were tested at CHUV. For axi-cel, five samples from a single patient were measured in parallel at two sites, our center and CHUV. Both sites used the ddPCR assay designed by us and described in this manuscript. For liso-cel, 12 samples were obtained from three patients treated at CHUV with a commercially available product and were measured at our center and CHUV using the ddPCR assay previously described by us [31].

## 5. Conclusions

We presented the design, implementation, and successful use of digital droplet PCR (ddPCR) for monitoring chimeric antigen receptor T-cell (CAR-T) expansion in patients with B-cell malignancies treated with different commercially available CAR-T products at our clinical center. Additionally, we applied this approach to quantify CAR-T mRNA expression, demonstrating a high correlation with DNA copy numbers and confirming active transgene expression. Our results highlighted the quality of ddPCR for CAR-T monitoring across multiple sample types, providing a sensitive, precise, and reproducible method to support the monitoring and management of CAR-T cell therapies in the clinical setting. We also described a patient cohort regarding peak level of CAR-T expansion (reached at a mean interval of 12 days post infusion for tisa-cel, 11 days for axi-cel, and 17 days for brexu-cel). More than half of all patients from our cohort were still alive 12 months after treatment. In the patients who reached the time point of 1 year after CAR-T therapy, 97% show measurable persistence of tisa-cel and 83% of brexu-cel; for axi-cel-treated patients, only 48% showed persistence.

## Figures and Tables

**Table 1:** Characteristics of patient cohort and CAR-T kinetics. The total number of patients receiving CAR-T therapy (no. of patients) and the peak of CAR-T expansion measured from blood samples analyzed in the routine setting with our ddPCR method, as average day of peak expansion and average copy number/µg DNA, is shown for each construct. Survival after 6 months and 1 year as well CAR-T persistence is included for all patients who have reached these milestones since the start of the therapy. The average day of CAR-T persistence from blood samples measured with ddPCR was calculated regardless of survival. * One patient was excluded because the last sample was available only 2 days after treatment due to death, ** another patient was excluded due to very late expansion after reinduction with glofitamab.

	Tisa-Cel	Axi-Cel	Brexu-Cel
**no. of patients**	73 (32 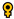 , 41 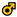 )	50 (22 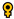 , 28 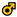 )	18 (7 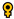 , 11 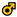 )
**peak CAR-T expansion**			
**day**	12 (2–84)	11 (5–26)	17 (5–61)
**average copies/µg DNA**	9733(265–127,942; n = 72 *)	12,375(37–91,575; n = 50)	7396(14–92,887; n = 17 **)
**survival**			
**at 6 months**	66% (48 of 73)	84% (42 of 50)	78% (14 of 18)
**at 1 year**	59% (43 of 73)	78% (39 of 50)	72% (13 of 18)
**persistence**			
**average [days]**	303 (n = 72)	187 (n = 50)	90 (n = 18)
**>1 year**	97% (33 of 34)	48% (10 of 21)	83% (5 of 6)

**Table 2:** Primer and probe sequences for ddPCR assays and oligonucleotides for Sanger sequencing.

**ddPCR Assays: Primers and Probes**		
tisa-cel (assay 1 own design [31])	CTL019 4-1BB F1	5′-GAAGATGGCTGTAGCTGCC-3′
	CTL019 CD3z R1	5′-GCTCCTGCTGAACTTCACTC-3′
	CTL019 4-1BBz probe	5′-FAM-GAAGAAGAAGAAGGAGGATGTGAACTG-BHQ1-3′
tisa-cel (assay 2, Milone et al. [32])	CTL019 4-1BB F2	5′-TGCCGATTTCCAGAAGAAGAAGAAG-3′
	CTL019 CD3z R3	5′-GCGCTCCTGCTGAACTTC-3′
	CTL019 4-1BBCD3z probe	5′-FAM-ACTCTCAGTTCACATCCTC-MGB-3′
liso-cel [31]	CTL019 4-1BB F1	5′-GAAGATGGCTGTAGCTGCC-3′
	CTL019 CD3z R4	5′-GCTTCTGCTGAACTTCACCC-3′
	CTL019 4-1BBz probe	5′-FAM-GAAGAAGAAGAAGGAGGATGTGAACTG -BHQ1-3′
*RPP30* [58]	RPP30 F	5′-AGATTTGGACCTGCGAGCG-3′
	RPP30 R	5′-GAGCGGCTGTCTCCACAAGT-3′
	RPP30 probe	5′-HEX-TTCTGACCTGAAGGCTCTGCGCG-BHQ1-3′
*ABL1*	ENF1003 short	5′-GAGATAACACTCTAAGCATAACTAAAG-3′
	ENR1063 short	5′-GTAGTTGCTTGGGACCCA-3′
	EnPr1043	5′-HEX-CCATTTTTGGTTTGGGCTTCACACCATT-BHQ1-3′
**Oligonucleotides for PCR and Sanger Sequencing**		
tisa-cel	CTL019 4-1BB SeqF	5′-ACGGGGCAGAAAGAAACTCC-3′
	CTL019 CD3z SeqR	5′-CTGTAGGCCTCCGCCATC-3′
axi-cel	Yescarta CD28 SeqF	5′-GGTGAGGAGTAAGAGGAGC-3′
	CTL019 CD3z SeqR	5′-CTGTAGGCCTCCGCCATC-3′
liso-cel	CTL019 4-1BB SeqF	5′-ACGGGGCAGAAAGAAACTCC-3′
	CTL019 CD3z SeqR2	5′-GATTCTGGCCCTGCTGGTAG-3′

## Data Availability

The original contributions presented in the study are included in the article and Supplementary Materials. Further inquiries can be directed to the corresponding author.

## Supplementary Materials

The following supporting information can be downloaded at: https://www.mdpi.com/article/10.3390/ijms25168556/s1.

## References

[1] Forsberg M.H., Das A., Saha K., Capitini C.M. (2018). The Potential of CAR T Therapy for Relapsed or Refractory Pediatric and Young Adult B-Cell ALL. Ther. Clin. Risk Manag..

[2] Munshi N.C., Anderson L.D., Shah N., Madduri D., Berdeja J., Lonial S., Raje N., Lin Y., Siegel D., Oriol A. (2021). Idecabtagene Vicleucel in Relapsed and Refractory Multiple Myeloma. N. Engl. J. Med..

[3] Berdeja J.G., Madduri D., Usmani S.Z., Jakubowiak A., Agha M., Cohen A.D., Stewart A.K., Hari P., Htut M., Lesokhin A. (2021). Ciltacabtagene Autoleucel, a B-Cell Maturation Antigen-Directed Chimeric Antigen Receptor T-Cell Therapy in Patients with Relapsed or Refractory Multiple Myeloma (CARTITUDE-1): A Phase 1b/2 Open-Label Study. Lancet.

[4] Charrot S., Hallam S. (2019). CAR-T Cells: Future Perspectives. HemaSphere.

[5] Neelapu S.S., Tummala S., Kebriaei P., Wierda W., Gutierrez C., Locke F.L., Komanduri K.V., Lin Y., Jain N., Daver N. (2018). Chimeric Antigen Receptor T-Cell Therapy-Assessment and Management of Toxicities. Nat. Rev. Clin. Oncol..

[6] Schuster S.J., Bishop M.R., Tam C.S., Waller E.K., Borchmann P., McGuirk J.P., Jäger U., Jaglowski S., Andreadis C., Westin J.R. (2019). Tisagenlecleucel in Adult Relapsed or Refractory Diffuse Large B-Cell Lymphoma. N. Engl. J. Med..

[7] Maude S.L., Laetsch T.W., Buechner J., Rives S., Boyer M., Bittencourt H., Bader P., Verneris M.R., Stefanski H.E., Myers G.D. (2018). Tisagenlecleucel in Children and Young Adults with B-Cell Lymphoblastic Leukemia. N. Engl. J. Med..

[8] Wang M., Munoz J., Goy A., Locke F.L., Jacobson C.A., Hill B.T., Timmerman J.M., Holmes H., Jaglowski S., Flinn I.W. (2020). KTE-X19 CAR T-Cell Therapy in Relapsed or Refractory Mantle-Cell Lymphoma. N. Engl. J. Med..

[9] Jacobson C.A., Chavez J.C., Sehgal A.R., William B.M., Munoz J., Salles G., Munshi P.N., Casulo C., Maloney D.G., de Vos S. (2022). Axicabtagene Ciloleucel in Relapsed or Refractory Indolent Non-Hodgkin Lymphoma (ZUMA-5): A Single-Arm, Multicentre, Phase 2 Trial. Lancet Oncol..

[10] Locke F.L., Miklos D.B., Jacobson C.A., Perales M.-A., Kersten M.-J., Oluwole O.O., Ghobadi A., Rapoport A.P., McGuirk J., Pagel J.M. (2022). Axicabtagene Ciloleucel as Second-Line Therapy for Large B-Cell Lymphoma. N. Engl. J. Med..

[11] San-Miguel J., Dhakal B., Yong K., Spencer A., Anguille S., Mateos M.-V., Fernández de Larrea C., Martínez-López J., Moreau P., Touzeau C. (2023). Cilta-Cel or Standard Care in Lenalidomide-Refractory Multiple Myeloma. N. Engl. J. Med..

[12] Rodriguez-Otero P., Ailawadhi S., Arnulf B., Patel K., Cavo M., Nooka A.K., Manier S., Callander N., Costa L.J., Vij R. (2023). Ide-Cel or Standard Regimens in Relapsed and Refractory Multiple Myeloma. N. Engl. J. Med..

[13] Laetsch T.W., Maude S.L., Rives S., Hiramatsu H. (2023). Three-Year Update of Tisagenlecleucel in Pediatric and Young Adult Patients With Relapsed/Refractory Acute Lymphoblastic Leukemia in the ELIANA Trial Clinical Trial Updates Abstract. J. Clin. Oncol..

[14] Schuster S.J., Tam C.S., Borchmann P., Worel N., McGuirk J.P., Holte H., Waller E.K., Jaglowski S., Bishop M.R., Damon L.E. (2021). Long-Term Clinical Outcomes of Tisagenlecleucel in Patients with Relapsed or Refractory Aggressive B-Cell Lymphomas (JULIET): A Multicentre, Open-Label, Single-Arm, Phase 2 Study. Lancet Oncol..

[15] Cappell K.M., Kochenderfer J.N. (2023). Long-Term Outcomes Following CAR T Cell Therapy: What We Know so Far. Nat. Rev. Clin. Oncol..

[16] Sun D., Shi X., Li S., Wang X., Yang X., Wan M. (2024). CAR-T Cell Therapy: A Breakthrough in Traditional Cancer Treatment Strategies (Review). Mol. Med. Rep..

[17] Trottmann M., Blozik E., Hilbig M., LoVerdi D., Pedruzzi M., Scherer T., Weiss M., Pletscher M., Meier N. (2023). Real-World Expenditures and Survival Time after CAR-T Treatment for Large B-Cell Lymphoma in Switzerland: A Retrospective Study Using Insurance Claims Data. Swiss Med. Wkly..

[18] Porter D.L., Hwang W.T., Frey N.V., Lacey S.F., Shaw P.A., Loren A.W., Bagg A., Marcucci K.T., Shen A., Gonzalez V. (2015). Chimeric Antigen Receptor T Cells Persist and Induce Sustained Remissions in Relapsed Refractory Chronic Lymphocytic Leukemia. Sci. Transl. Med..

[19] Frigault M.J., Maus M.V. (2020). State of the Art in CAR T Cell Therapy for CD19+ B Cell Malignancies. J. Clin. Investig..

[20] Wittibschlager V., Bacher U., Seipel K., Porret N., Wiedemann G., Haslebacher C., Hoffmann M., Daskalakis M., Akhoundova D., Pabst T. (2023). CAR T-Cell Persistence Correlates with Improved Outcome in Patients with B-Cell Lymphoma. Int. J. Mol. Sci..

[21] Heini A.D., Bacher U., Kronig M.-N., Wiedemann G., Novak U., Zeerleder S., Mansouri Taleghani B., Daskalakis M., Pabst T. (2022). Chimeric Antigen Receptor T-Cell Therapy for Relapsed Mantle Cell Lymphoma: Real-World Experience from a Single Tertiary Care Center. Bone Marrow Transplant..

[22] Hays A., Durham J., Gullick B., Rudemiller N., Schneider T. (2023). Bioanalytical Assay Strategies and Considerations for Measuring Cellular Kinetics. Int. J. Mol. Sci..

[23] DePriest B.P., Vieira N., Bidgoli A., Paczesny S. (2021). An Overview of Multiplexed Analyses of CAR T-Cell Therapies: Insights and Potential. Expert Rev. Proteom..

[24] Berger S.C., Fehse B., Rubio M.-T., Kröger N., Gribben J., Chabannon C., Yakoub-Agha I., Einsele H. (2022). Immune Monitoring. The EBMT/EHA CAR-T Cell Handbook.

[25] Galli E., Viscovo M., Fosso F., Pansini I., Di Cesare G., Iacovelli C., Maiolo E., Sorà F., Hohaus S., Sica S. (2024). Unlocking Predictive Power: Quantitative Assessment of CAR-T Expansion with Digital Droplet Polymerase Chain Reaction (DdPCR). Int. J. Mol. Sci..

[26] García-Calderón C.B., Sierro-Martínez B., García-Guerrero E., Sanoja-Flores L., Muñoz-García R., Ruiz-Maldonado V., Jimenez-Leon M.R., Delgado-Serrano J., Molinos-Quintana Á., Guijarro-Albaladejo B. (2023). Monitoring of Kinetics and Exhaustion Markers of Circulating CAR-T Cells as Early Predictive Factors in Patients with B-Cell Malignancies. Front. Immunol..

[27] Hay K.A., Hanafi L.A., Li D., Gust J., Liles W.C., Wurfel M.M., López J.A., Chen J., Chung D., Harju-Baker S. (2017). Kinetics and Biomarkers of Severe Cytokine Release Syndrome after CD19 Chimeric Antigen Receptor–Modified T-Cell Therapy. Blood.

[28] Locke F.L., Ghobadi A., Jacobson C.A., Miklos D.B., Lekakis L.J., Oluwole O.O., Lin Y., Braunschweig I., Hill B.T., Timmerman J.M. (2019). Long-Term Safety and Activity of Axicabtagene Ciloleucel in Refractory Large B-Cell Lymphoma (ZUMA-1): A Single-Arm, Multicentre, Phase 1–2 Trial. Lancet Oncol..

[29] Neelapu S.S. (2019). Managing the Toxicities of CAR T-cell Therapy. Hematol. Oncol..

[30] Stein A.M., Grupp S.A., Levine J.E., Laetsch T.W., Pulsipher M.A., Boyer M.W., August K.J., Levine B.L., Tomassian L., Shah S. (2019). Tisagenlecleucel Model-Based Cellular Kinetic Analysis of Chimeric Antigen Receptor–T Cells. CPT Pharmacomet. Syst. Pharmacol..

[31] Pabst T., Joncourt R., Shumilov E., Heini A., Wiedemann G., Legros M., Seipel K., Schild C., Jalowiec K., Mansouri Taleghani B. (2020). Analysis of IL-6 Serum Levels and CAR T Cell-Specific Digital PCR in the Context of Cytokine Release Syndrome. Exp. Hematol..

[32] Milone M.C., Fish J.D., Carpenito C., Carroll R.G., Binder G.K., Teachey D., Samanta M., Lakhal M., Gloss B., Danet-Desnoyers G. (2009). Chimeric Receptors Containing CD137 Signal Transduction Domains Mediate Enhanced Survival of T Cells and Increased Antileukemic Efficacy in Vivo. Mol. Ther..

[33] Mika T., Maghnouj A., Klein-Scory S., Ladigan-Badura S., Baraniskin A., Thomson J., Hasenkamp J., Hahn S.A., Wulf G., Schroers R. (2020). Digital-Droplet PCR for Quantification of CD19-Directed CAR T-Cells. Front. Mol. Biosci..

[34] Wang H., Du X., Chen W.H., Lou J., Xiao H.L., Pan Y.M., Chen H., An N., Zhang Q.X. (2018). Establishment of a Quantitative Polymerase Chain Reaction Assay for Monitoring Chimeric Antigen Receptor T Cells in Peripheral Blood. Transplant. Proc..

[35] Badbaran A., Berger C., Riecken K., Kruchen A., Geffken M., Müller I., Kröger N., Ayuk F.A., Fehse B. (2020). Accurate In-Vivo Quantification of Cd19 Car-t Cells after Treatment with Axicabtagene Ciloleucel (Axi-Cel) and Tisagenlecleucel (Tisa-Cel) Using Digital Pcr. Cancers.

[36] Raje N., Berdeja J., Lin Y., Siegel D., Jagannath S., Madduri D., Liedtke M., Rosenblatt J., Maus M.V., Turka A. (2019). Anti-BCMA CAR T-Cell Therapy Bb2121 in Relapsed or Refractory Multiple Myeloma. N. Engl. J. Med..

[37] Fehse B., Badbaran A., Berger C., Sonntag T., Riecken K., Geffken M., Kröger N., Ayuk F.A. (2020). Digital PCR Assays for Precise Quantification of CD19-CAR-T Cells after Treatment with Axicabtagene Ciloleucel. Mol. Ther. Methods Clin. Dev..

[38] Kalos M., Levine B.L., Porter D.L., Katz S., Grupp S.A., Bagg A., June C.H. (2011). T Cells with Chimeric Antigen Receptors Have Potent Antitumor Effects and Can Establish Memory in Patients with Advanced Leukemia. Sci. Transl. Med..

[39] Food and Drug Administration U.S. Food and Drug Administration Center for Biologics Evaluation and Research (2020). Long Term Follow-Up after Administration of Human Gene Therapy Products Guidance for Industry.

[40] Lou Y., Chen C., Long X., Gu J., Xiao M., Wang D., Zhou X., Li T., Hong Z., Li C. (2020). Detection and Quantification of Chimeric Antigen Receptor Transgene Copy Number by Droplet Digital PCR versus Real-Time PCR. J. Mol. Diagn..

[41] Kochenderfer J.N., Feldman S.A., Zhao Y., Xu H., Black M.A., Morgan R.A., Wilson W.H., Rosenberg S.A. (2009). Construction and Preclinical Evaluation of an Anti-CD19 Chimeric Antigen Receptor. J. Immunother..

[42] Heini A.D., Bacher U., Porret N., Wiedemann G., Legros M., Stalder Zeerleder D., Seipel K., Novak U., Daskalakis M., Pabst T. (2022). Experiences with Glofitamab Administration Following CAR T Therapy in Patients with Relapsed Mantle Cell Lymphoma. Cells.

[43] Messerli C., Wiedemann G., Porret N., Nagler M., Seipel K., Jeker B., Novak U., Zeerleder S., Bacher U., Pabst T. (2023). Correlation of Peripheral Chimeric Antigen Receptor T-Cell (CAR-T Cell) MRNA Expression Levels with Toxicities and Outcomes in Patients with Diffuse Large B-Cell Lymphoma. Turk. J. Hematol..

[44] Sugimoto H., Chen S., Minembe J.P., Chouitar J., He X., Wang H., Fang X., Qian M.G. (2021). Insights on Droplet Digital PCR–Based Cellular Kinetics and Biodistribution Assay Support for CAR-T Cell Therapy. AAPS J..

[45] Shah N.N., Qin H., Yates B., Su L., Shalabi H., Raffeld M., Ahlman M.A., Stetler-Stevenson M., Yuan C., Guo S. (2019). Clonal Expansion of CAR T Cells Harboring Lentivector Integration in the CBL Gene Following Anti-CD22 CAR T-Cell Therapy. Blood Adv..

[46] Nobles C.L., Sherrill-Mix S., Everett J.K., Reddy S., Fraietta J.A., Porter D.L., Frey N., Gill S.I., Grupp S.A., Maude S.L. (2020). CD19-Targeting CAR T Cell Immunotherapy Outcomes Correlate with Genomic Modification by Vector Integration. J. Clin. Investig..

[47] Sheih A., Voillet V., Hanafi L.A., DeBerg H.A., Yajima M., Hawkins R., Gersuk V., Riddell S.R., Maloney D.G., Wohlfahrt M.E. (2020). Clonal Kinetics and Single-Cell Transcriptional Profiling of CAR-T Cells in Patients Undergoing CD19 CAR-T Immunotherapy. Nat. Commun..

[48] Fraietta J.A., Nobles C.L., Sammons M.A., Lundh S., Carty S.A., Reich T.J., Cogdill A.P., Morrissette J.J.D., DeNizio J.E., Reddy S. (2018). Disruption of TET2 Promotes the Therapeutic Efficacy of CD19-Targeted T Cells. Nature.

[49] Yamamoto S., ichi Matsumoto S., Goto A., Ugajin M., Nakayama M., Moriya Y., Hirabayashi H. (2020). Quantitative PCR Methodology with a Volume-Based Unit for the Sophisticated Cellular Kinetic Evaluation of Chimeric Antigen Receptor T Cells. Sci. Rep..

[50] Awasthi R., Pacaud L., Waldron E., Tam C.S., Jäger U., Borchmann P., Jaglowski S., Foley S.R., Van Besien K., Wagner-Johnston N.D. (2020). Tisagenlecleucel Cellular Kinetics, Dose, and Immunogenicity in Relation to Clinical Factors in Relapsed/Refractory DLBCL. Blood Adv..

[51] Locke F.L., Neelapu S.S., Bartlett N.L., Siddiqi T., Chavez J.C., Hosing C.M., Ghobadi A., Budde L.E., Bot A., Rossi J.M. (2017). Phase 1 Results of ZUMA-1: A Multicenter Study of KTE-C19 Anti-CD19 CAR T Cell Therapy in Refractory Aggressive Lymphoma. Mol. Ther..

[52] Ayuk F.A., Berger C., Badbaran A., Zabelina T., Sonntag T., Riecken K., Geffken M., Wichmann D., Frenzel C., Thayssen G. (2021). Axicabtagene Ciloleucel in Vivo Expansion and Treatment Outcome in Aggressive B-Cell Lymphoma in a Real-World Setting. Blood Adv..

[53] Monfrini C., Stella F., Aragona V., Magni M., Ljevar S., Vella C., Fardella E., Chiappella A., Nanetti F., Pennisi M. (2022). Phenotypic Composition of Commercial Anti-CD19 CAR T Cells Affects In Vivo Expansion and Disease Response in Patients with Large B-Cell Lymphoma. Clin. Cancer Res..

[54] Gagelmann N., Bishop M., Ayuk F., Bethge W., Glass B., Sureda A., Pasquini M.C., Kröger N. (2024). Axicabtagene Ciloleucel versus Tisagenlecleucel for Relapsed or Refractory Large B Cell Lymphoma: A Systematic Review and Meta-Analysis. Transplant. Cell. Ther..

[55] Fürst D., Neuchel C., Neagoie A., Amann E., Rode I., Krauss A., Schrezenmeier H., Wais V., Döhner H., Viardot A. (2022). Monitoring the In-Vivo Expansion and Persistence of CAR-T Cells as a Tool to Help Decision Making in Patients with Aggressive B-Cell Lymphoma. Blood.

[56] Bachy E., Le Gouill S., Di Blasi R., Sesques P., Manson G., Cartron G., Beauvais D., Roulin L., Gros F.X., Rubio M.T. (2022). A Real-World Comparison of Tisagenlecleucel and Axicabtagene Ciloleucel CAR T Cells in Relapsed or Refractory Diffuse Large B Cell Lymphoma. Nat. Med..

[57] He H.J., Stein E.V., DeRose P., Cole K.D. (2018). Limitations of Methods for Measuring the Concentration of Human Genomic DNA and Oligonucleotide Samples. Biotechniques.

[58] Härmälä S.K., Butcher R., Roberts C.H. (2017). Copy Number Variation Analysis by Droplet Digital PCR. Methods Mol. Biol..

[59] Beillard E., Pallisgaard N., van der Velden V.H.J., Bi W., Dee R., van der Schoot E., Delabesse E., Macintyre E., Gottardi E., Saglio G. (2003). Evaluation of Candidate Control Genes for Diagnosis and Residual Disease Detection in Leukemic Patients Using “real-Time” Quantitative Reverse-Transcriptase Polymerase Chain Reaction (RQ-PCR)—A Europe against Cancer Program. Leukemia.

